# Efficient DEM simulations of railway ballast using simple particle shapes

**DOI:** 10.1007/s10035-022-01274-y

**Published:** 2022-09-13

**Authors:** Bettina Suhr, Klaus Six

**Affiliations:** Virtual Vehicle Research GmbH, Inffeldgasse 21/A, A-8010, Graz, Austria

**Keywords:** Railway ballast, DEM modelling, Simple particle shapes, Calibration

## Abstract

For complex shaped materials, computational efficiency and accuracy of DEM models are usually opposing requirements. In the literature, DEM models of railway ballast often use very complex and computationally demanding particle shapes in combination with very simple contact laws. In contrast, this study suggests efficient DEM models for railway ballast using simple particle shapes together with a contact law including more physical effects. In previous works of the authors, shape descriptors, calculated in a shape analysis of two types of ballast, were used to construct simple particle shapes (clumps of three spheres). Using such a shape in DEM simulations of compression and direct shear tests, accurate results were achieved only when the contact law included additional physical effects e.g. edge breakage. A parametrisation strategy was developed for this contact law comparing DEM simulations with the measurements. Now, all the constructed simple particle shapes are parametrised allowing to study their suitability and relating their shape descriptors to those of railway ballast. The most suitable particle shapes consist of non-overlapping spheres, thus have a high interlocking potential, and have lowest sphericity and highest convexity values. In a micromechanical analysis of the four best performing shapes, three shapes show similar behaviour on the bulk and the micro-scale, while one shape differs clearly on the micro-scale. This analysis shows, which shapes can be expected to produce similar results in DEM simulations of other tests/load cases. The presented approach is a step towards both efficient and accurate DEM modelling of railway ballast.

**Graphic abstract:**

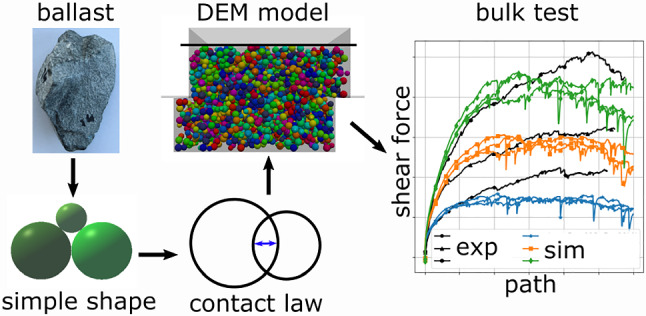

## Introduction

In the discrete element modelling of granular materials, particle shape and size representation plays an important role. Together with particle contact modelling and model calibration it determines the accuracy and reliability of the DEM model.

Shape modelling is usually the crucial factor for computational efficiency. Therefore, from computational point of view it is interesting to ask: Is it possible to build an accurate DEM model using simple particle shapes (which have potentially a high inaccuracy of shape modelling) and combine them with a suitable contact law and a proper parametrisation? This study aims to contribute to answering this question using the example of railway ballast.

As the single ballast stones have very complex shapes, including sharp edges and corners, particle shape modelling is frequently addressed in the literature on DEM modelling of railway ballast. Using 2D or 3D scanner data of ballast stones, rigid clumps of spheres are constructed in [5–7, 11, 14, 19]. These particle shape models are highly detailed and usually consist of a high number of spheres (above 10 in [11] and above 50 in [6]). This high accuracy in shape modelling comes at the price of a high computational effort, which increases not only with the number of spheres, but also with decreasing sphere radii, needed to construct sharp corners or edges. Polyhedra are also used for modelling railway ballast; e.g. 3D-scanned ballast stones are used in [9, 10, 15, 16, 30] to construct polyhedral DEM particles, with respect to certain shape descriptors. While these models look very realistic, their computational effort is usually higher than that of clumps of spheres (dependent on the actual shape and the simulation software used) and it increases with the number of corners being modelled. In the literature, also approaches can be found which use potential particles for modelling railway ballast, see [1, 8]. Ahmed et al. [1] present a method to manually adapt the shape of a potential particle to the shape of a ballast stone. Almost all studies mentioned above use highly detailed particle shapes to model ballast stones. The exceptions are Laryea et al. [13], Chen et al. [2] and Coetzee [3], where simple particle shape models are applied, but only qualitative, not quantitative agreement between simulations and experiments are obtained. For DEM modelling of railway ballast, its common to combine complex particle shapes with very simple contact laws with purely elastic material behaviour not accounting for relevant physical phenomena like edge breakage, etc. For computational efficiency, it would therefore be preferable to reduce the complexity in particle shape modelling and to increase the complexity in contact modelling.

The effect of particle shape is investigated in the literature intensively. In most studies, particle shape is varied (mostly with respect to certain shape descriptors) using the same contact law and parameters for all shapes and the simulated bulk behaviour is analysed. In the review paper [4], several of such studies are cited.

When a particular granular material is to be modelled via DEM, a comparison using different particle shapes should be accompanied by a parametrisation for each used shape. Such studies are rare in literature and application specific. An example is Coetzee [3], where crushed rock was modelled using both manually and automatically generated clumps of spheres. A parametrisation was conducted for each clump shape and the resulting DEM models were compared in validation tests. The simplest clump shape used (consisting of two spheres) performed slightly worse, while all other clump shapes together with their individual parameters could pass the validation tests.

The question how accurate particle shape of a given material must be modelled in DEM and how much of “shape inaccuracies” can be compensated by an individual parametrisation remains open. Although the different particle shapes in [3] could be parametrised to give a similar bulk behaviour, the different particle shapes and parameters used are likely to cause differences in the packing at micromechanical level.

**Fig. 1:**
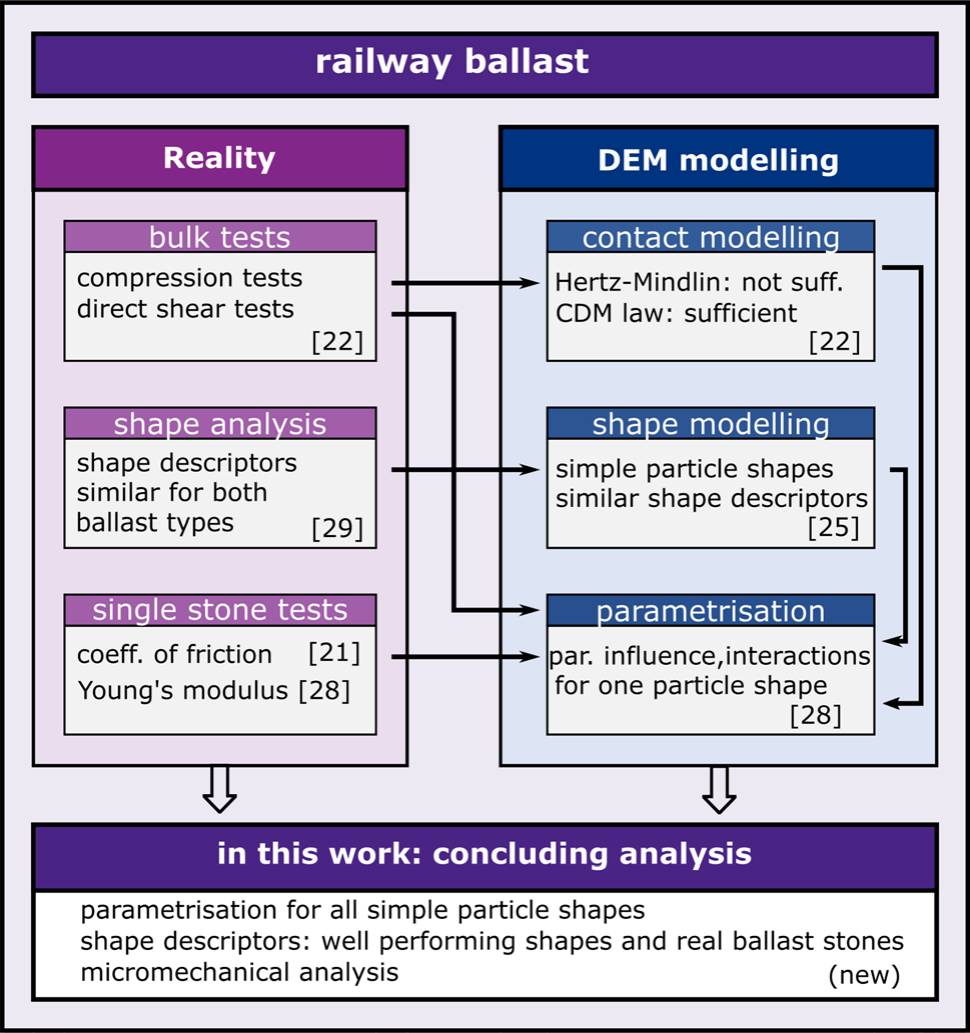
Sketch of previous and current work on railway ballast

This work tries to contribute to answer the above question and continues previous works of the authors on DEM modelling of railway ballast. Figure 1 shows a graphical overview of previous works, including particle shape modelling, contact modelling and parametrisation methods on the DEM side and on the experimental side bulk tests, a shape analysis and single stone measurements. In experiments conducted on two types of railway ballast, named Calcite and Kieselkalk, a very similar behaviour was seen in the direct shear test, while clear differences occurred in the uniaxial compression test.

A shape analysis with these two types of railway ballast was conducted, Suhr et al. [29], investigating 3D scanner data of ballast stones for several shape descriptors. No difference between the two types of ballast were found regarding flatness, elongation, roughness, sphericity, convexity index or a newly developed curvature-based angularity index. Thus, the observed difference in bulk material behaviour between Calcite and Kieselkalk are unlikely to be caused by differences in particle shape.

The knowledge on ballast shape was used in [25] to systematically construct simple particle shapes, which have similar shape descriptors as the real stones. Aiming for computational efficiency, clumps of three spheres were investigated and by analysing their packing behaviour, 20 simple particle shapes were found, which pack at the same porosities as Calcite and Kieselkalk ballast in experiments.

Contact modelling was addressed in [22], comparing simulations with measured compression and direct shear tests. In the experiments, both types of ballast showed a very similar behaviour in the direct shear test, but clear differences could be seen in the uniaxial compression test. In [22], using a simple particle shape, clumps of three spheres, and applying the classical Hertz–Mindlin contact law, it was not possible to bring simulation results in good accordance with experimental measurements. However, this could be achieved, when the Conical Damage Model (CDM), developed in [8], was used as contact law. In the CDM law the elastic part of the material behaviour is modelled via the Hertz law. Additionally, a kind of ideal plasticity is is introduced to model damage at a contact (e.g. to take into account edge breakage). The CDM law has four parameters (two more than the classical Hertz–Mindlin law). Using the CDM law and applying a trial and error approach with quantitative error measures, for both types of ballast, one set of parameters could be found, such that simulations using simple particle shapes gave results in good agreement with the measurements.

In [28], a parametrisation strategy for the CDM law was developed. Wherever possible, measured values were used to define ranges of parameters. Measurements of the Young’s modulus of both types of ballast were conducted, Suhr et al. [27]. Single stone measurements on the coefficient of friction of both types of ballast showed a high amount of scatter, see [21]. As the CDM law involves four parameters, the problem of possible parameter ambiguity was investigated in detail in [28]. High parameter ambiguity means that different (non neighbouring) parameter sets give the same (or similar) simulation results. It is questionable whether a DEM model parametrised under such conditions to one type of experiment, can give reliable predictions of different experiments or applications. Using the method of virtual calibration, for all seven considered test cases the calibration was successful, but some parameter ambiguity with respect to *E* and $\mu$ was observed. Knowledge on the measured Young’s modulus of ballast single stones, the influence of the parameters on the simulation results and the possible parameter ambiguity, the DEM model was parametrised to the measurement data of compression and direct shear tests. Also, suggestions for reducing the computational effort of the parametrisation were given and tested successfully.

This work will use the knowledge gained from previous works to finish a complete analysis of DEM modelling of one type of railway ballast. 20 different simple particle shapes will be considered for the parametrisation to Kieselkalk ballast. While problems occurred for some particle shapes, 10 shapes are successfully parametrised to the experimental data. The shape descriptors of these shapes are compared to those of the ballast stones, to check for possible relations. A micromechanical analysis reveals that considering different particle shapes with their own set of parameters, the internal states of the packings can differ, although the bulk response is similar to the experimental one.

This work is organised as follows: in Sect. 2 the results of the conducted shape analysis and the constructed simple particle shapes are briefly summarised. In Sect. 3, details on the DEM simulations are given: the used contact law and the sample generation and pre-compaction. In Sect. 4 the used parametrisation method, a summary of the parametrisation results and the micromechanical analysis are presented. Finally, in Sect. 5 conclusions are drawn. Full details on the obtained results of the parametrisation are given in the Appendix A.

## Summary of shape analysis and particle shape modelling

In [29], a shape analysis of the two types of ballast, Calcite and Kieselkalk, was conducted. This analysis was based on 3D scans of 25 stones each. Well-established shape descriptors, such as elongation, *e*, flatness, *f*, sphericity, $\psi$, convexity index, *c*, were evaluated. These shape descriptors are defined below in Eqs. (1a)–(1d), denoting by *L*, *I*, *S* the longest, intermediate and shortest axes of the particle’s minimum bounding box, *V* the particle’s volume and *A* the particle’s surface area.

1a $$\begin{aligned} e& = I/L \end{aligned}$$

1b $$\begin{aligned} f& = S/I \end{aligned}$$

1c $$\begin{aligned} \psi& = \root 3 \of {36 \pi V^2} / A \end{aligned}$$

1d $$\begin{aligned} c& = V(\text{ convex } \text{ hull}) / V(\text{ particle}) \end{aligned}$$

Regarding these shape descriptors no difference could be seen between both types of railway ballast: Calcite and Kieselkalk.

Moreover, three different curvature based angularity indices were compared. Only a newly introduced angularity index, the scaled Willmore energy, gave reasonable results in analytic tests, application to scanned data of sharp stones as well as artificially smoothed versions of the scanned stones. No difference could be seen between Calcite and Kieselkalk ballast with respect to this angularity index. As clumps of spheres will be used as DEM shapes, here the calculation of angularity makes no sense. Therefore, angularity will not be considered further.

**Fig. 2:**
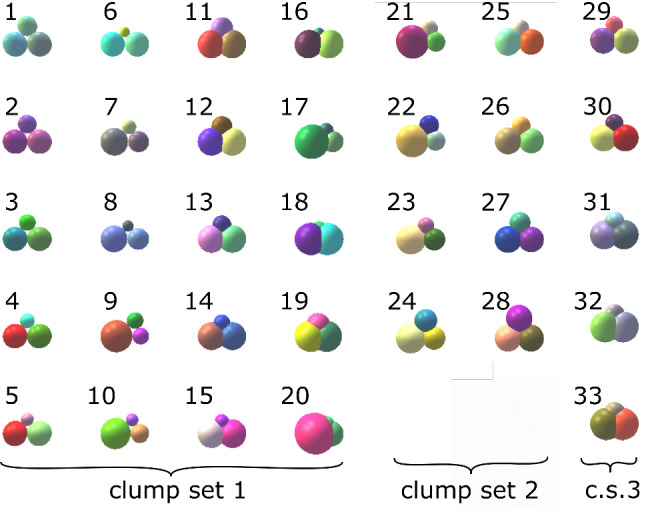
Constructed simple particle shapes, ordered in three clump sets, compare [25]

In [25], simple particle shapes where constructed, which consisted of clumps of three spheres (for reason of computational efficiency). In contrast to many other studies, the clumps did not aim to have a similar shape as the real particles but to have similar shape descriptors. They were numbered from 1 to 31 and were organised in three different clump sets, compare Fig. 2. In clump set 1, shapes 1–10 consist of non-overlapping spheres (particle with hole), while shapes 11 to 21 have a high overlap (no hole). In clump shape 2, the spheres were arranged to have a low overlap and no hole (the three spheres intersect exactly in one point). In clump set 3, one particle shape is chosen and the amount of overlap is varied in finer steps. The packing behaviour of all clump shapes was analysed and from initially 31 simple particle shapes 20 were found, which pack at the same porosities as Calcite and Kieselkalk ballast (from experiments): shapes 1–10 (non-overlapping spheres) and shapes 21 to 30 (low amount of overlap).

Obviously, the constructed clumps of spheres lack the angularity of real ballast stones. However, they are non-convex, which is an important property of ballast, and also allows for interlocking. Clumps of spheres can be arranged to obtain particles with higher or lower “surface roughness”, Soltanbeigi et al. [18]. Roth and Jaeger [17] and Irazabal et al. [12] describe particles with corrugated surfaces or cavities between overlapped spheres with the expression “geometric friction”, which is also known to affect the packing porosity. To quantify this mentioned “geometric friction” of a clump, a so called “clump roughness angle”, $\delta$ was introduced in [25]. In addition to the clump roughness angle, the clumps are further characterised by the so called “member size difference”, $s_d$, which is the difference between the maximal and minimal radius of the spheres forming the clump.

**Fig. 3:**
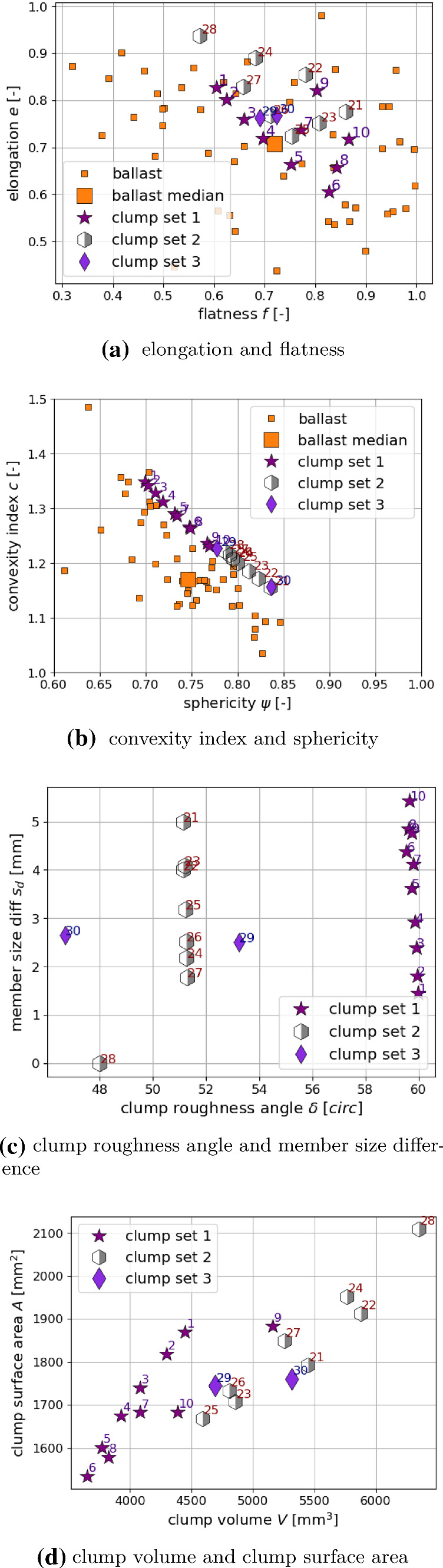
Shape descriptors of the chosen clumps (1–10 and 21–30), compared to those real ballast stones when applicable, compare [25]

The shape descriptors of these chosen clumps (1–10 and 21–30) are plotted in Fig. 3. Where possible the shape descriptors of the real ballast stones are also shown; as no difference could be seen between Calcite and Kieselkalk, the corresponding values are merged. Elongation and flatness values of the real ballast stones cover a wide range of values in Fig. 3a. The chosen clumps also cover quite a big part of their possible range. The porosity $\phi$ was used for choosing suitable clumps and in [25] neither elongation nor flatness showed a strong correlation with $\phi$. In Fig. 3b, *c* and $\psi$ of the chosen clumps are plotted together with the values of the real ballast stones. A strong correlation of the porosity $\phi$ with *c* and $\psi$ was seen in [25]. All chosen clumps have $c\ge 1.15$ and $\psi \le 0.84$, while all rejected clumps have smaller *c* and larger $\psi$ values (not shown in the plot). The range of $(c, \psi )$ values of the clump shapes matches well with those of the real ballast stones. Thus, when a different material is to be modelled via simple particle shapes, matching the convexity and sphericity might be a good first step. In Fig. 3c, clump roughness angle $\delta$ and member size difference $s_d$ are plotted. Both quantities are specific to the clump modelling and therefore no comparison to the ballast stones is possible. While the $s_d$ values of the chosen clumps cover nearly the complete range, it is apparent that the chosen clumps have all $\delta \ge 45^\circ$, while all rejected clumps have smaller $\delta$ values (not shown in the plot). This result was expected, as $\delta$ correlated strongly with the porosity $\phi$ (which was used to choose the clumps) but $s_d$ did not show this correlation with $\phi$. Finally, Fig. 3d shows the volume and surface area of the chosen clumps. Although *V* showed a strong correlation with the porosity, chosen clump shapes and rejected ones could not be separated by volume. This is in contrast to the other shape descriptors, which do correlate strongly with the porosity, i.e. $c, \psi , \delta$. The volume and surface area of the clumps is smaller than the ones of the real stones, due to DEM modelling conventions, i.e. longest size of the particle and sample size have a ratio of 10. Therefore, no comparison is shown.

## DEM simulation details

**Fig. 4:**
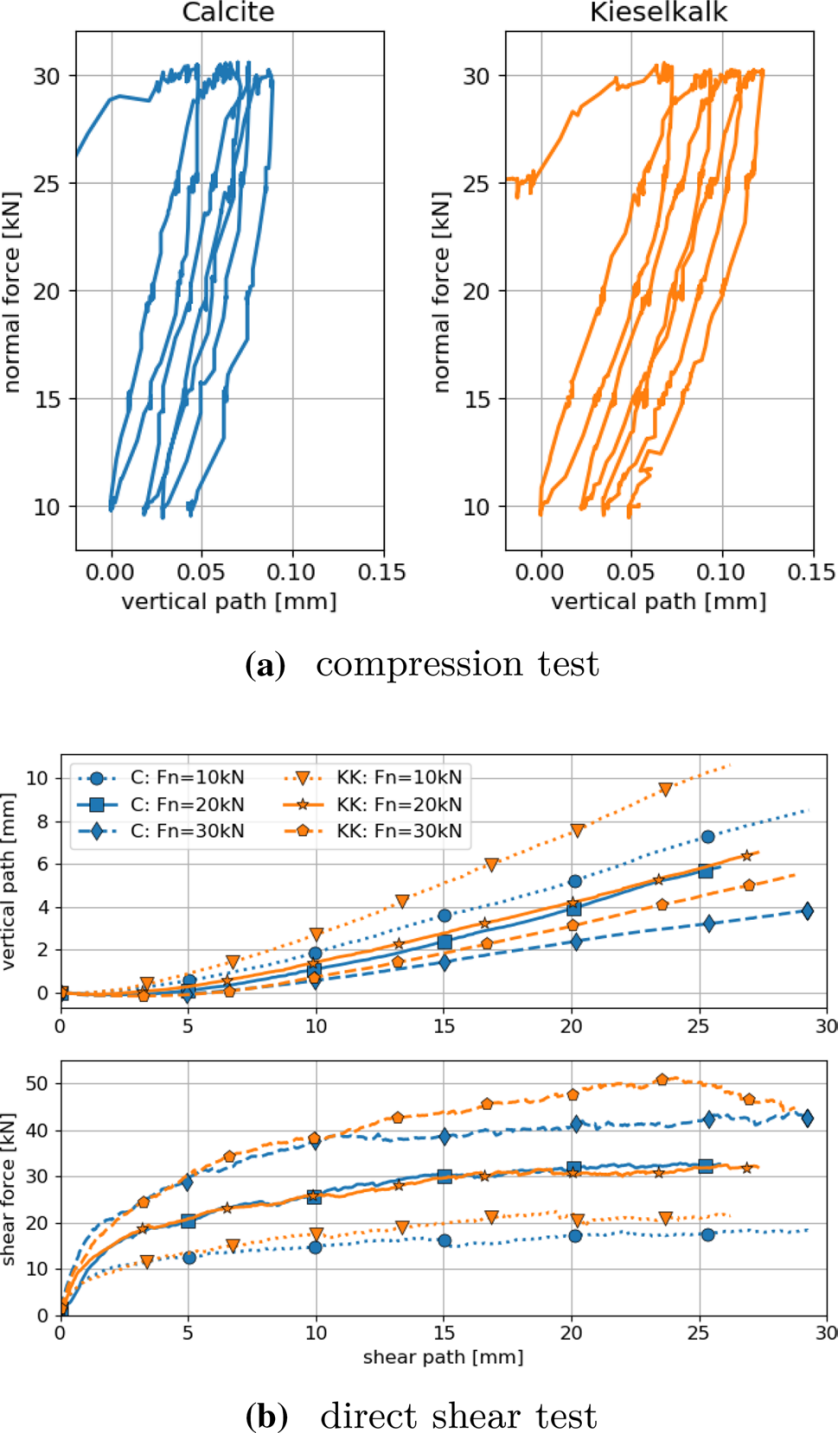
Experimental results of compression and direct shear test for Calcite (C) and Kieselkalk (KK) ballast

For all DEM simulations in this work the software YADE [31] will be used. It is Open-Source and utilises the soft contact approach together with explicit integration in time. DEM simulations of uniaxial compression and direct shear tests will be conducted and compared to experimental results. The experimental results are shown in Fig. 4, see [28] for details and post processing of the data.

### Used contact model: the CDM law

In this work, the CDM contact law will be used based on the experiences gained in [22, 23] with the simulation of the compression and direct shear test. The CDM law, developed in [8], is an extension of the Hertz–Mindlin model, where a kind of ideal plasticity is is introduced to model damage at a contact (e.g. to take into account edge breakage), which turned out to be necessary to describe the observed behaviour in the experiments.

For two contacting spheres (sphere wall contact is analogue), the DEM software calculates the geometric overlap $\delta _{\text{DEM}}$. In the CDM law, this geometric overlap is split in an elastic part, $\delta _{el}$, and a plastic part, $\delta _{pl}$: $\delta _{\text{DEM}} = \delta _{el} + \delta _{pl}$, where, $\delta _{pl}$ is initialised with zero when the contact is created. The normal force is calculated with the Hertz law using only the elastic part of the overlap:

2 $$\begin{aligned} F_n = \frac{4}{3} E^* \sqrt{R} \left( \delta _{el}\right) ^\frac{3}{2} \; , \end{aligned}$$

where $E^*$ is the equivalent Young’s modulus and *R* is the current radius in the contact which is initialized with the equivalent radius $R^*$. According to Hertzian theory, the maximal stress at the contact, $\sigma _0$, can be calculated as

3 $$\begin{aligned} \sigma _0 = \frac{2 E^*}{ \pi } \sqrt{ \frac{\delta _{el}}{R} } \; . \end{aligned}$$

In the CDM law, a pseudo maximal compressive strength $\sigma _\text {{max}}$ is assumed. If $\sigma _0 >\sigma _\text {{max}}$, then the stress is too high for the material to be carried and damage/plastic yielding occurs. The spheres in contact are thought to flatten locally, thus *R* and $\delta _{pl}$ increase such that $\sigma _0 = \sigma _\text {{max}}$. The relation between $\delta _{pl}$ and *R* are derived in [8] such that

4 $$\begin{aligned} \delta _{pl} = (R - R^*) \beta \; , \end{aligned}$$

where the material parameter $\beta$ relates to an opening angle of a conical asperity $\alpha$ as: $\beta = \frac{1-\sin (\alpha )}{ \sin (\alpha )}$. From the limitation of the stress $\sigma _0 = \sigma _\text {{max}}$ in the plastic case, it follows:

5 $$\begin{aligned} R = \frac{ \delta _{\text{DEM}}+ R^* \beta }{ \left( \frac{ \sigma _\text {{max}}\pi }{2 E^*}\right) ^2 + \beta } \; . \end{aligned}$$

With this equation it is possible to solve the model accurately without the need of an iterative procedure, see [23] for details. Note that the actual radii of the spheres in the DEM model remains unchanged, just the radius of the contact area, *R*, increases in the contact law. In this formulation of the CDM law, the calculation of the tangential force remains unchanged from the Hertz–Mindlin law. In total the CDM law has four parameters: *E*, $\sigma _\text {{max}}$, $\alpha$ and $\mu$.

### Sample generation and pre-compaction

As mentioned before, the simple particle shapes constructed in [25] will be used: shapes numbers 1–10 (clumps of non-overlapping spheres) and 21–30 (clumps of spheres with low amount of overlap).

Sample generation and pre-compaction is done using the method developed in [28] and will be summarised here briefly. The samples were generated using the CDM law in a two step procedure. For all particle shapes, a given set of parameters was used. Using a rainfall procedure, particles were filled in the shear box and after settling all particles above the box were deleted. Varying the initial friction coefficient, $\mu _\text {{initial}}$, the mass of the sample in the box was achieved to be similar to the mass of the experimental specimens (approx. 26 kg for Kieselkalk ballast). This initial configuration was saved to a file before pre-compaction.

For the second step of pre-compaction, this file was loaded and the contact parameters were set to their final values, except $\mu = \mu _\text {{initial}}$. A normal load was applied on the sample until a porosity of 0.445 was reached (median values of experimental specimens), then the sample was unloaded. The normal load necessary to reach the target porosity was strongly dependent on the used parameters and the particle shape. However, this load was in all cases lower than the maximal load applied in the compression test. After unloading, the coefficient of friction $\mu$ was set to its final value. This pre-compaction procedure caused a considerable yielding of contacts, which lead to an increase of the contacts’ radii *R*. This procedure of sample generation and pre-compaction ensures that simulations using different sets of parameters and particle shapes all have the same mass and a similar initial porosity (while their internal state $R, \delta _{pl}$ will differ). Different methods of pre-compaction will lead to different internal states, which can be expected to influence the simulated bulk behaviour and thus the parametrisation result. Due to the high number of conducted simulations in this study, the chosen pre-compaction strategy had to be fast to simulate. Therefore, the application of cyclic loading (similar as in the experimental pre-compaction) was prohibitive.

In the specimen generation, the particle shapes 1–10 (non-overlapping spheres) have considerably lower values of $\mu _\text {{initial}}$ (between 0.2 and 0.5) than the remaining shape numbers 21 to 30, consisting of overlapping spheres ($\mu _\text {{initial}}$ around 0.6). This can be explained with the shape’s different clump roughness angles $\delta$, see Fig. 3c. Also, the clump roughness angle should be a measure for the shapes “geometric friction”, which is known to influence the packing porosity [12, 17].

## Parametrisation of DEM models for different particle shapes

**Fig. 5:**
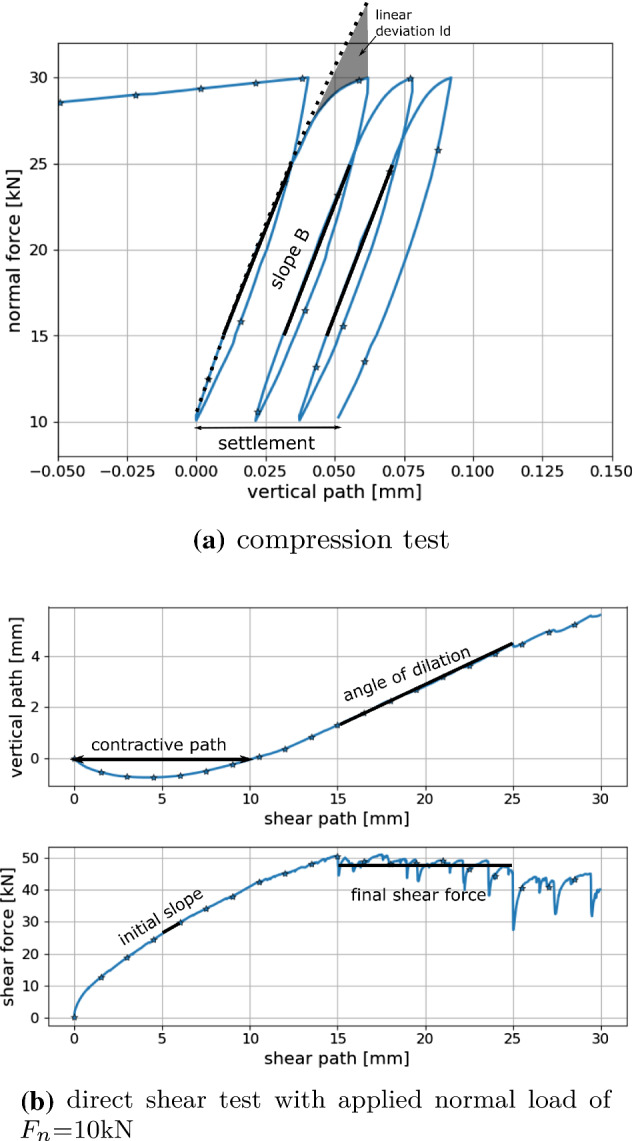
Simulation results for compression and direct shear test, including sketches of characteristics, compare [28]

The parametrisation of the DEM models to the compression and direct shear tests is conducted using the method described in [28]. In the parametrisation step the parameters of the CDM law are searched, $E, E/\sigma _\text {{max}}, \alpha , \mu$, which bring the simulation results close to the experimental ones. For both experiments conducted, so-called characteristics were defined: settlement, slope and linear deviation for the compression test and angle of dilation, contractive path, initial slope and final shear force for the direct shear test, compare Fig. 5. These characteristics were used to define a cost function for the parametrisation. The general idea for the construction of the cost function was to consider the deviation of a simulation results from the median value of the experimental results and to set this in relation to the scattering seen in the experiments. The characteristics were evaluated for all experimental results to calculate the median value and the maximal deviation of the experiments from their median value for each considered characteristic. For a simulation result for one parameter set $p_i = (E, E/\sigma _\text {{max}}, \alpha , \mu )$ the characteristics can be evaluated and a cost function for each characteristic was defined as the difference between simulation and experimental median value of characteristic divided by the maximal deviation of the experimental values. Thus, a value below one means that the simulation result lies in the range of the deviation of the experiments for this characteristic. From these cost functions for a single characteristics, a cost function for the compression test was defined $\varepsilon ^{odo}(p_i)$ using simulation results for three different initial configurations. In a similar manner a cost function for the direct shear test was defined, $\varepsilon ^{shear}(p_i)$, which takes into account DEM simulations of three levels of applied normal load for three different initial configurations. A combined cost function $\varepsilon (p_i)$ is defined as the mean value of the cost functions for compression and direct shear test, see [28] for details.

As a first step of the parametrisation, compression tests are simulated using parameters belonging to a full Design of Experiments (DoE) plan. The levels of the parameters are given in in Table 1. For the Young’s modulus *E*, the parameter levels were chosen according to the measurements values of Kieselkalk ballast, see [28]. For the remaining parameters, the ranges and levels were defined manually after evaluating first simulation results.

According to the parametrisation strategy, outlined in [28], the simulations of the compression test were evaluated for the full DoE plan. For those parameter sets, which gave low values of the cost function $\varepsilon ^{odo}$, simulation of the direct shear test were conducted. After evaluating the cost function for the direct shear test, $\varepsilon ^{shear}$ and the overall cost function $\varepsilon$, parameter sets with lowest errors were chosen for repetition simulations (with different initial samples).

**Table 1:** Parameter levels of the DoE plan for shapes 1–10 and shapes 21–30

Parameter					
*Shapes 1–10*					
*E* [GPa]	50				
$E/\sigma _\text {{max}}$ [–]	150	125	100	75	
$\alpha \,[^\circ ]$	79	81	83	85	87
$\mu$ [–]	0.4	0.5			
*Shapes 21–30*					
*E* [GPa]	50	67			
$E/\sigma _\text {{max}}$ [–]	150	125	100	75	
$\alpha \,[^\circ ]$	79	81	83	85	87
$\mu$ [–]	0.6	0.7			

As discussed in [28], scatter is a problem in the parametrisation process on different levels. Results from simulations using the same parameter sets but different initial configuration do scatter with respect to the characteristics (to different extents). Moreover, the simulation results of the directs shear test itself show a relatively high scatter in the shear force, which sometimes leads to kinks in the dilation curve. The observed scatter is clearly an obstacle for a fast and precise parametrisation and prevents the use of optimisation methods. Moreover, in virtual calibration test conducted in [28], some parameter ambiguity with respect to *E* and $\mu$ was observed. These difficulties and limitations have to be kept in mind, when the parametrisation results for different particle shapes are discussed.

**Fig. 6:**
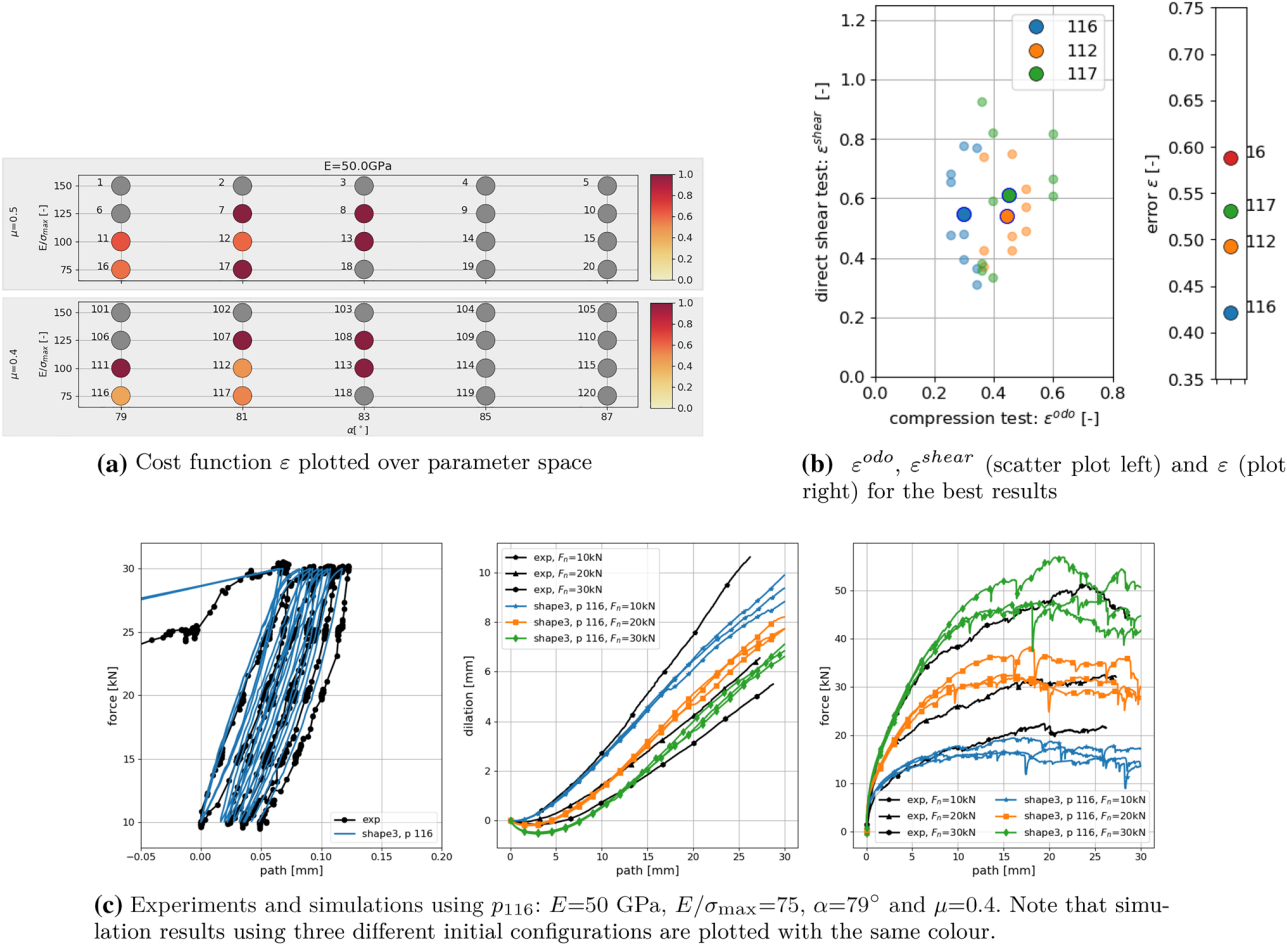
Shape 3: results of parametrisation using three initial configurations for simulations

### Parametrisation results

Figur 6 shows the results of the parametrisation for shape 3. In Fig. 6a, the overall cost function $\varepsilon$ is plotted over the parameter space of the used DoE plan, shown together with the numbering of the parameters. Note that this numbering is chosen such that $p_1$ to $p_{20}$ belong to $\mu = 0.5$, while the corresponding parameter sets belonging to $\mu = 0.4$ are denoted with $p_{101}$ to $p_{120}$. The coloured points are parameter sets, where simulations of both compression and direct shear test are conducted for three initial configurations; all other points are shown in grey (thus only results for the best parameter sets are shown). The colour corresponds to the value of the cost function $\varepsilon$. In this plot, the mentioned parameter ambiguity can be seen for $\mu$. In Fig. 6b in the plot on the right, the 4 best parameter sets are shown with respect to the overall error $\varepsilon$. Also shown in Fig. 6b (left) is a scatter plot of the three best results $p_{116}, p_{112}, p_{117}$ over $\varepsilon ^{odo}$, $\varepsilon ^{shear}$. Small and bright symbols correspond to single simulations belonging to the three different initial configurations; simulations of one initial configuration give one vale of $\varepsilon ^{odo}$ for the compression test and three values of $\varepsilon ^{shear}$ belonging to the three levels of applied normal load in direct shear test. Thus, for one parameter set nine points are plotted in the Figure. For clarity, the mean value of these nine points is plotted in the Figure as well using large symbols. In this plot, the scattering of the results with respect to different initial configurations can be seen. Finally, Fig. 6c shows a comparison of the experimental results of the compression and the direct shear test for Kieselkalk ballast and the simulation results obtained with parameter set $p_{116}$.

**Fig. 7:**
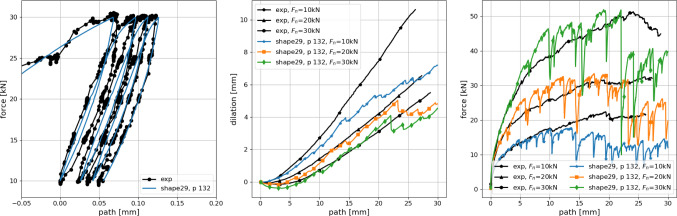
Shape 29: results of parametrisation compared to experiments. DEM simulations use parameter set $p_{132}$: *E* = 67 GPa, $E/\sigma _\text {{max}}$ = 100, $\alpha$ = 81$^\circ$ and $\mu$ = 0.7

During the parametrisation process of shapes 21 to 29 severe problems occurred. As an example Fig. 7 shows the best results of simulations (only one initial configuration) for shape 29. In the direct shear test, for all levels of applied normal load the shear stress ramps up and the drops by about 50%. The resulting dilation curve is not linear any more and to calculate the angle of dilation makes little sense. As mentioned before, shapes 21 to 29 have a lower clump roughness angle than shapes 1–10, compare Fig. 3c. As a result, they need higher values of $\mu _\text {{initial}}$ for specimen generation and consequently higher values for $\mu$. Together with the shapes’ geometry this higher $\mu$ values lead to the problematic behaviour in the direct shear test: a higher friction coefficient allows the development of higher shear stresses. The massive drops in the shear stress are attended by sharp increases in the number of sliding contacts. Compared to shapes 1–10, the lower clump roughness angles have less potential to stop these sliding events by geometric interlocking. Due to these problems occurring, the parametrisation of shapes 21–29 was skipped.

**Fig. 8:**
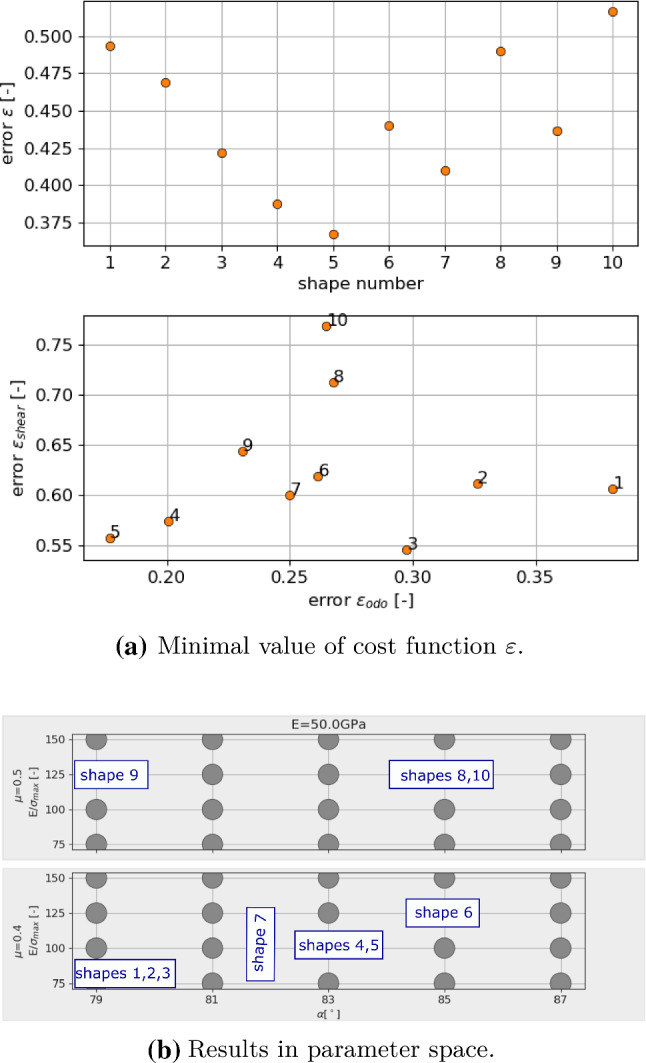
Summary of parametrisation results for shapes 1–10

All remaining shapes 1–10 consist of clumps of non-overlapping spheres. For these shapes the parametrisation to Kieselkalk ballast was successful. The full details on the parametrisation results can be found in Appendix A. Here, a summary of the obtained values of the cost functions and the parameter sets is given in Fig. 8. Remember that cost functions are constructed with respect to scattering of experimental results such that cost function values below 1 are considered acceptable. In the upper part of Fig. 8a, the values of the cost function $\varepsilon$ for shapes 1–10 are shown. For all shapes the cost function values lie below 0.52 and from the path-force curves shown in Appendix A all of them can be considered acceptable for the DEM simulation of Kieselkalk ballast. Looking at the differences among the 10 shapes in Fig. 8a, it is surprising that the obtained overall error $\varepsilon$ reduced continuously from shapes 1 to 5. The lower part of Fig. 8a shows the errors in compression test, $\varepsilon _{odo}$, and shear test, $\varepsilon _{shear}$, for the different shapes. Comparing both plots, the reduction of the overall error $\varepsilon$ for shapes 1 to 5 results from a reduction in the error of the compression test $\varepsilon _{odo}$ for these shapes. The error in the compression test is more difficult to see in a visual inspection than the error in the direct shear test. For example, the obtained results for shapes 5 and 3 (see Appendix A, Figs. 14a, 13c) look similar, because their error in the directs shear test $\varepsilon _{shear}$ is similar. The advantage of a parametrisation based on error definitions, compared to the pure visual comparison, is the ability to quantify the better fit of shape 5 in the compression test. One has to keep in mind that the results obtained in the conducted parametrisation do depend on the definition of the cost functions and on a possible weighting of the single contributions to $\varepsilon _{odo}$ and $\varepsilon _{shear}$.

In Fig. 8b the obtained results of the parametrisation are shown in the parameter space. Note that shape 7 was parametrised in previous work, Suhr et al. [28], and the parameter set obtained ( $E = 50$ GPa, $E/\sigma _\text {{max}} = 100$, $\alpha$ = 82$^\circ$ and $\mu$ = 0.45) does not fit to the grid of parameters used here. For some shapes the same parameter sets are obtained: shapes 1, 2 and 3, shapes 4 and 5, shapes 8, 10 (shape 6 has the same parameters with a lower $\mu$ value). The discussion of the results in parameter space will be continued at the end of the next subsection, taking into account the values of their shape descriptors.

### Shape descriptors of particle shapes

**Fig. 9:**
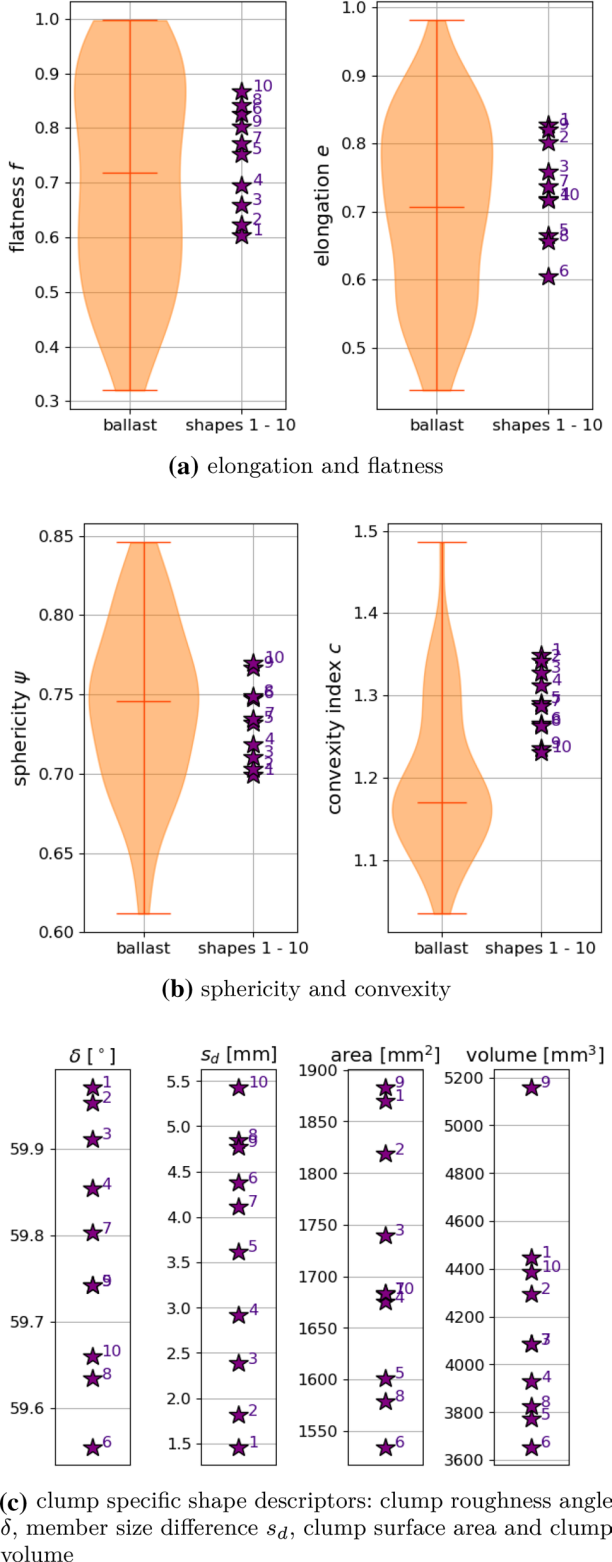
Shape descriptors of successfully parametrised particle shapes. Where possible a comparison to real ballast stones is shown

In this subsection, the shape descriptors of the successfully parametrised particle shapes, 1–10, will be compared to those of the declined shapes and those of real ballast stones. Fig. 9 shows a detailed plot of the shape descriptors of ballast stones and shapes 1–10. The shape descriptor values of the ballast stones are shown as violin plot, which is similar to a box plot with minimum, maximum and median value but also includes the probability density of the underlying data. For both elongation and flatness values of shapes 1–10 lie well within the range of the values of the real ballast stones. However, this is also the case for the declined shapes (can be seen in Fig. 3). Elongation and flatness are thus not suitable to distinguish between shapes that could be successfully parametrised and those how could not.

On the contrary, such a distinction is possible with respect to sphericity and convexity. The sphericity values of shapes 1–10 lie between 0.7 and 0.77, while all declined shapes had higher sphericity values. The median value of the ballast stones’ sphericity is shortly below 0.75 and is thus in the range of values of shapes 1–10. The convexity values of shapes 1–10 lie between 1.23 and 1.35, while all declined shapes had lower convexity values. The values of shapes 1–10 are clearly higher than the median value of the ballast stones, but still lower than the maximal measured value of nearly 1.5. The declined shapes reach values below the ballast median value.

The most dominant influence seen in the parametrisation is seen for the clump roughness angle. Shapes 1–10 are all non-overlapping clumps and thus have the highest potential for interlocking. Their clump roughness angles are all round 60$^\circ$. All declined shapes had lower clump roughness values. In contrast, the member size difference of accepted and declined shapes cover the same range of values. Regarding clump surface area and clump volume, shapes 1–10 had mostly smaller values than the declined shapes, but no sharp distinction was possible.

Summarising, for the construction of simple particle shapes for DEM modelling of railway ballast the key influence factors are the clump roughness angle, sphericity and convexity, while clump surface area and volume also show some influence. Shapes with the highest clump roughness angles, lowest sphericity, highest convexity values and lowest surface area and volume gave the best results.

It is interesting to relate the shape descriptors of shapes 1–10 to the parametrisation results shown in Fig. 8b. Those shapes, which give result in the same parameter sets, are also similar with respect to their values of sphericity $\psi$ and convexity index *c*, see Fig. 9. In general, with an increase in shape numbers, *c* decreases and $\psi$ increases almost monotonically. These trends correspond roughly to a trend in the parameter space in Fig. 8b: with increasing shape numbers, $E/\sigma _\text {{max}}$ increases, $\alpha$ increases as well as $\mu$. Thus, considering the bulk behaviour of the DEM models using the different shapes, to some extent a decrease in the convexity index and an increase in sphericity of the shapes can be compensated by increasing the parameters $E/\sigma _\text {{max}}$, $\alpha$ and $\mu$. An exception to the mentioned trend is shape 9, whose best parameter set is not neighbouring the results of the other shapes. However, in Appendix A it can be seen that the second best parameter set for this shape is neighbouring the parameter set of shapes 8 and 10.

Two conclusions enhance the trust in the parametrisation method: first, for the considered similar shapes the obtained parameter sets are neighbouring. Second, their shape descriptors can be connected roughly to the identified parameters.

### Micromechanical analysis

In this subsection, it will be investigated, if micromechanical differences exist between the particle shapes, which give the best result in the parametrisation: shapes 5, 4, 7 and 3. They have a similar bulk behaviour: close to the experimental one. Remembering the previous section, shapes 4 and 5 have the same parameter set, the parameter set of shape 7 differs slightly, while the parameter set of shape 3 differs more. Both particle shape and the used parameters will influence the micromechanical behaviour. Please note that the coordination numbers of a packing strongly depends on the chosen particle shape representation. Due to the chosen simple particle shapes, the coordination numbers reported in this study are considerably higher than those reported in literature for DEM simulations of railway ballast.

**Fig. 10:**
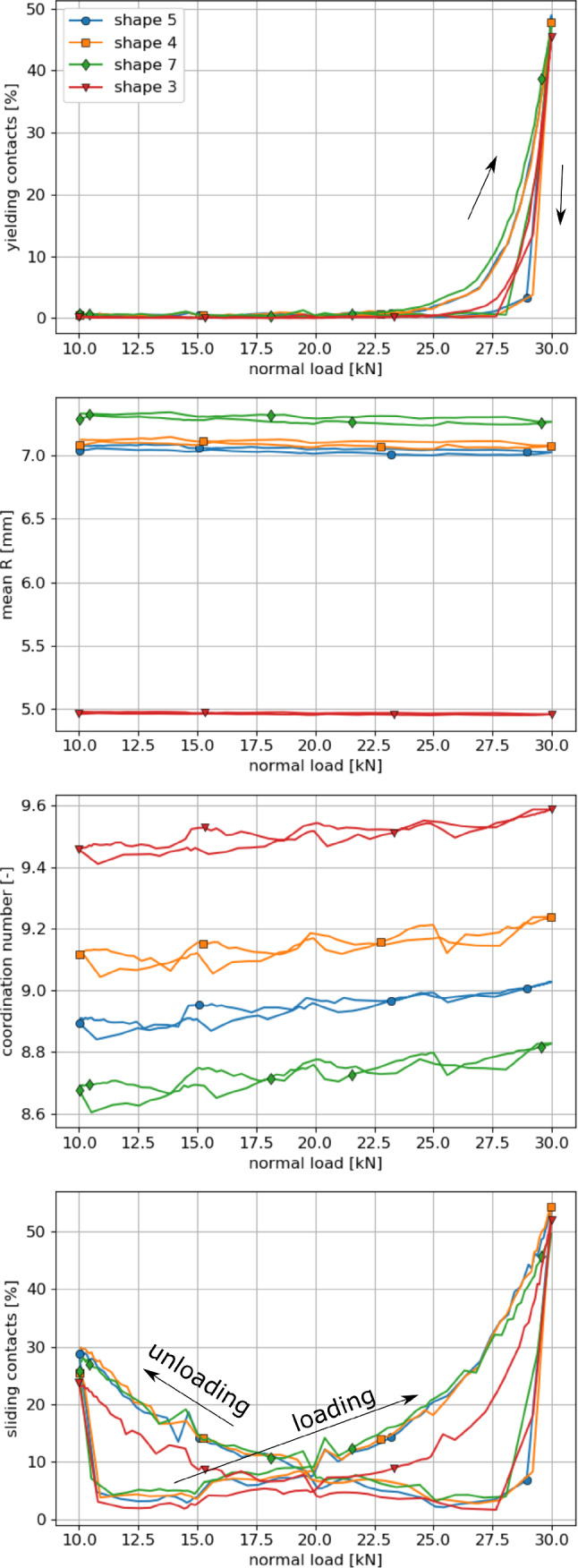
Compression test: micromechanical analysis of best shapes: 5, 4, 7, 3

**Fig. 11:**
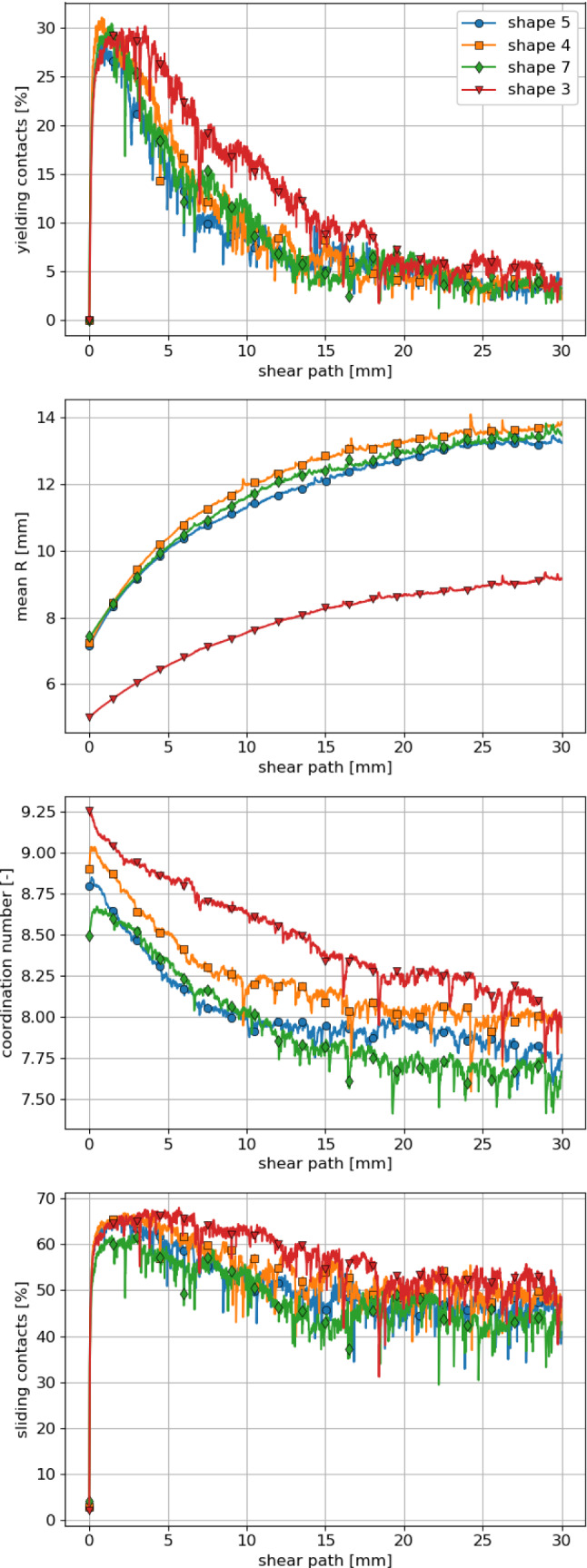
Direct shear test at 20 kN: micromechanical analysis of best shapes: 5, 4, 7, 3

For the compression test, the specimens are compared during the second loading cycle. To characterise the internal state, the following quantities are plotted over the applied normal force in Fig. 10: the percentage of yielding contacts, the mean value of *R*, the coordination number and the percentage of sliding contacts. In the compression test, contact yielding plays an important role, which can be seen at the relatively high amount of yielding contacts. The behaviour of the four particle shapes is very similar: in the loading phase below 25 kN little till no yielding contacts occurs. With increasing load the percentage of yielding contacts rises up to almost 50% and drops sharply to zero in the unloading phase. The plotted mean values of *R* remain nearly constant during this loading cycle. During the loading cycle, contacts are lost and new ones are created, some of which yield. In total, the mean value of *R* remains mostly constant for the single shapes. The big differences seen between the shapes (between shape 3 and the remaining shapes) stem from the pre-compaction phase and the loading to the first load maximum (remember the parameter set of shape 3 differs more from the other shapes’ parameter sets).

The coordination number increases slowly during the loading and decreases under unloading as expected. Clear differences between particle shapes can be seen, which remain nearly constant during the considered time.

For the percentage of sliding contacts the following behaviour is seen. At the load minimum about 25% of the contacts slide. Under loading this percentage reduces sharply to roughly 5% and increases then slowly with increasing load to its maximum at about 50%. During unloading the percentage of sliding contact drops fast below 10% and increases then with reducing normal to about 30% at the load minimum. The behaviour of shapes 5, 4, and 7 is almost identical, while the one of shape 3 shows some deviations.

For a micromechanical analysis of the direct shear test, the same quantities are plotted in Fig. 11 over the shear path. The Figure shows simulations belonging to an applied normal load of 20 kN. The percentage of yielding contacts is around 30% at the beginning of the shear test and decrease until about 5% yielding contacts are reached at 20 mm shear path. With continued shearing this percentage stays almost constant. The results for shapes 5, 4, and 7 are nearly the same, while shape 3 shows a slightly decayed decrease of the yielding contacts.

The mean value of *R* is nearly same for shapes 5, 4 and 7 and again much lower for shape 3, which can be related to the differing parameter set of this shape. For all shapes, a strong increase in the mean *R* is seen until 20 mm shear path followed by a moderate increase with further shearing. At shearing contacts, the geometric interlocking has to be overcome first. In some cases, this will be accompanied by yielding, probably with strong increases in *R*. Due to dilation the sample gets looser over the shear path and the increase in *R* slows down.

The coordination number reduces during shearing for all shapes. At the first half of the shear path this reduction is stronger than for the second half. As in the compression test, shape 3 shows the highest coordination number, followed by shape 4, 5, and 7. Compared to the compression test, the coordination numbers are lower, which can be explained by the fully unloading of the end of the compression test.

Naturally, the percentage of sliding contacts is very high in the direct shear test. At the beginning of the test, values between 60 and 65% are seen which reduce to values between 40 and 50% after about 15 mm shear path and remain mostly constant during further shearing. The behaviour of the different shapes is very similar, with results for shape 3 slightly higher than for the other shapes.

Finally, the micromechanical behaviour of the four shapes 5, 4, 7 and 3 in the compression as well as in the direct shear test gives nearly identical results for the percentage of yielding contacts and the percentage of sliding contacts. Small differences between the shapes are seen in the coordination number for both tests. However, clear differences can be seen in the mean value of *R*: while shapes 5, 4 and 7 have very similar values, these are lower for shape 3. Thus, shapes 5, 4 and 7, who have similar parameter sets, show a similar bulk and micromechanical behaviour in the compression and direct shear test. This finding gives confidence that DEM models using these shapes for simulations of other tests/load cases would also produce similar results. However, this is less likely to be the case for shape 3, which has a similar bulk behaviour but differs on micromechanical scale, i.e. *R*. When different particle shapes are compared for the simulation of a certain material, a micromechanical analysis is advisable.

## Conclusions

This work is the last part of a series of papers on DEM modelling of railway ballast. Two types of ballast named Calcite and Kieselkalk were tested for their bulk behaviour: in uniaxial compression tests Calcite showed a higher stiffness than Kieselkalk, while in a direct shear tests both types of ballast gave very similar results [22]. To investigate the reason of this difference further measurements were conducted. Based on 3D scans of single ballast stones a shape analysis was conducted in [29]. For the considered shape descriptors elongation, flatness, angularity index, sphericity, convexity index and roughness no differences were found between Calcite and Kieselkalk. Single stone measurements on the coefficient of friction resulted in similar values for both types of ballast [21]. In contrast, by measuring the Young’s modulus clearly higher values were obtained for Calcite than for Kieselkalk [27]. This result was a plausible explanation for the observed differences in the stiffness in the compression test.

All information from the experimental investigations were then used to build DEM models for both types of ballast. Aiming at computational efficiency, simple particle shapes (clumps of three spheres) were constructed in [25], which tried to approximate the shape descriptors of the ballast stones (in contrast to approximating the complex shapes of the stones). Investigating packing behaviour, 20 possible particle shapes were identified for modelling the two considered ballast types. Particle contact modelling was addressed in [22] using simple particle shapes: while it was not possible to parametrise the Hertz–Mindlin law with compression and direct shear tests, this was achieved using the CDM law.

The CDM law has four parameters, thus it is a huge task to parametrise DEM models for 20 particle shapes to the compression and direct shear tests. As a preliminary step, Suhr et al. [27] investigated the influence of the parameters on the simulation results for one simple particle shape. A virtual calibration step was used to investigate possible parameter ambiguity. High parameter ambiguity means that different (non neighbouring) parameter sets give the same (or similar) simulation results. It is questionable whether a DEM model parametrised under such conditions to one type of experiment, can give reliable predictions of different experiments or applications. For all considered test cases the calibration was successful, but some parameter ambiguity with respect to *E* and $\mu$ was observed. Using knowledge on the ballast single stones measurements, the DEM model was parametrised to the measurement data of compression and direct shear tests for this particle shape. Also, suggestions for reducing the computational effort of the parametrisation were given and tested successfully for a second particle shape.

In this study, the developed parametrisation method was applied to the 20 simple particle shapes to model Kieselkalk ballast. For some particle shapes, problems arose during parametrisation. Nevertheless, for particle shapes 1–10 the parametrisation was successful. The cost function used for the parametrisation was constructed such that values below 1 are considered acceptable. For shapes 1–10 the cost function values lie below 0.52 and thus all 10 shapes are considered acceptable for DEM simulation of Kieselkalk ballast.

Shapes 1–10 consist of non-overlapping spheres and have the highest value of the clump roughness angle, indicating high potential for interlocking. From all constructed particle shapes, they have the lowest values of sphericity, the highest convexity index and mostly lower values of surface area and volume. In comparison to the shape descriptors of the ballast stones, the sphericity values of shapes 1–10 lie around the ballast stones’ median value. The convexity values of shapes 1–10 are clearly higher than the ballast median value, while they are still in the range of the ballast’s convexity values. Regarding flatness and elongation, some of shapes 1–10 are close the the ballasts median values, while others are farer away.

In the parametrisation method, neighbouring parameter sets are obtained for the considered shapes, which are similar with respect to sphericity and convexity index and the clump roughness angle, which is nearly identical for shapes 1–10. Moreover, in DEM simulations using the different shapes, to some extent a decrease in the convexity index and an increase in sphericity of the shapes can be compensated by increasing the parameters $E/\sigma _\text {{max}}$, $\alpha$ and $\mu$. These findings enhance the trust in the parametrisation method.

Considering the four shapes that gave the best results in the parametrisation, shapes 5, 4, 7 and 3, a micromechanical analysis was conducted. In the compression and the direct shear tests shapes 5, 4, and 7 showed nearly identical results regarding the percentage of yielding contacts, mean value of *R* and percentage of sliding contacts. For the coordination number, small differences were seen between the shapes. Shape 3 also showed similar results regarding the percentage of yielding or sliding contacts and the coordination number. However, the mean values of *R* were considerably lower for this shape. This finding increases the confidence that DEM models using shapes 5, 4 or 7 for simulations of other tests/load cases would also produce similar results, as their bulk and micromechanical behaviour is similar. When different particle shapes are compared for the simulation of a certain material, a micromechanical analysis is advisable to detect cases like shape 3 (with a similar bulk but different micromechanical behaviour).

The presented results show that computationally efficient simple particle shapes combined with the CDM law are promising for DEM modelling of railway ballast. In contrast to high fidelity shape models, such simple particle shapes aim to capture key aspects of shape, such as interlocking potential. For a final assessment, validation tests with different load cases would be necessary.

## Figures and Tables

**Fig. 12:**
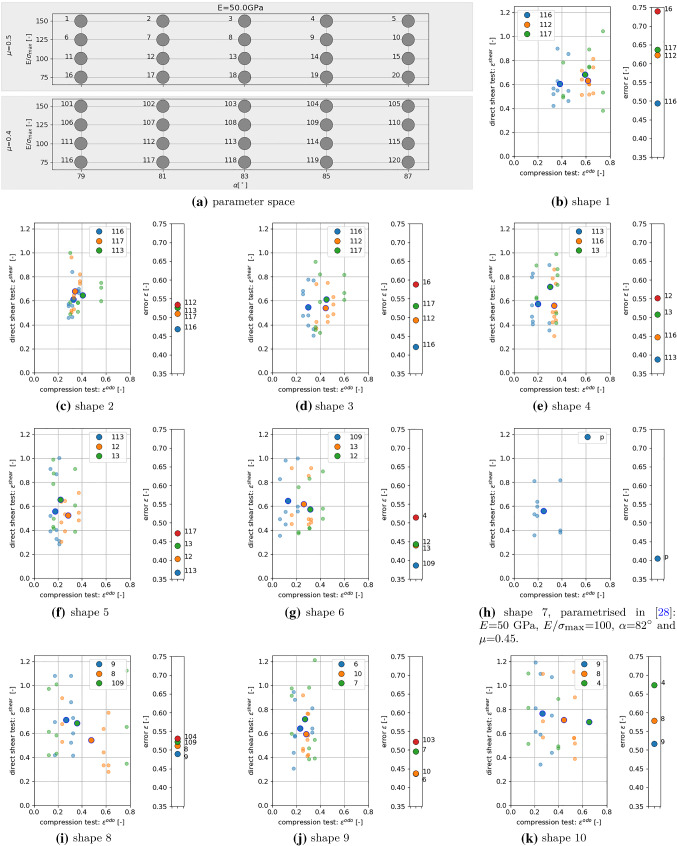
Scatter of results of parametrisation for Kieselkalk ballast: $\varepsilon ^{odo}$, $\varepsilon ^{shear}$ and $\varepsilon$

**Fig. 13:**
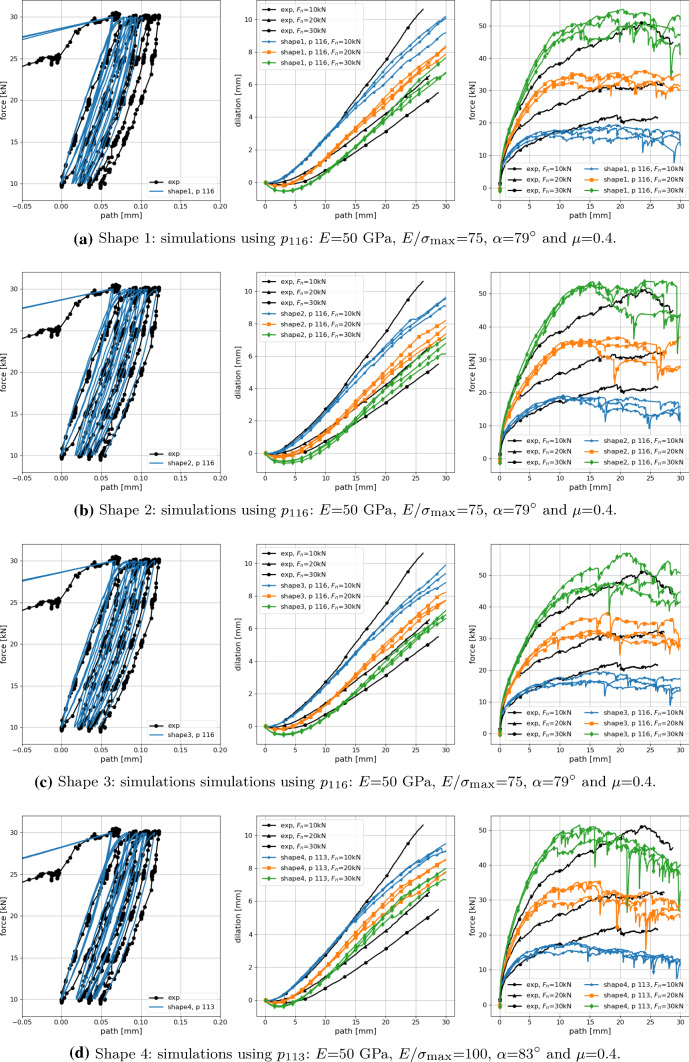
Shapes 1 to 4: results of parametrisation in comparison to Kieselkalk experiments

**Fig. 14:**
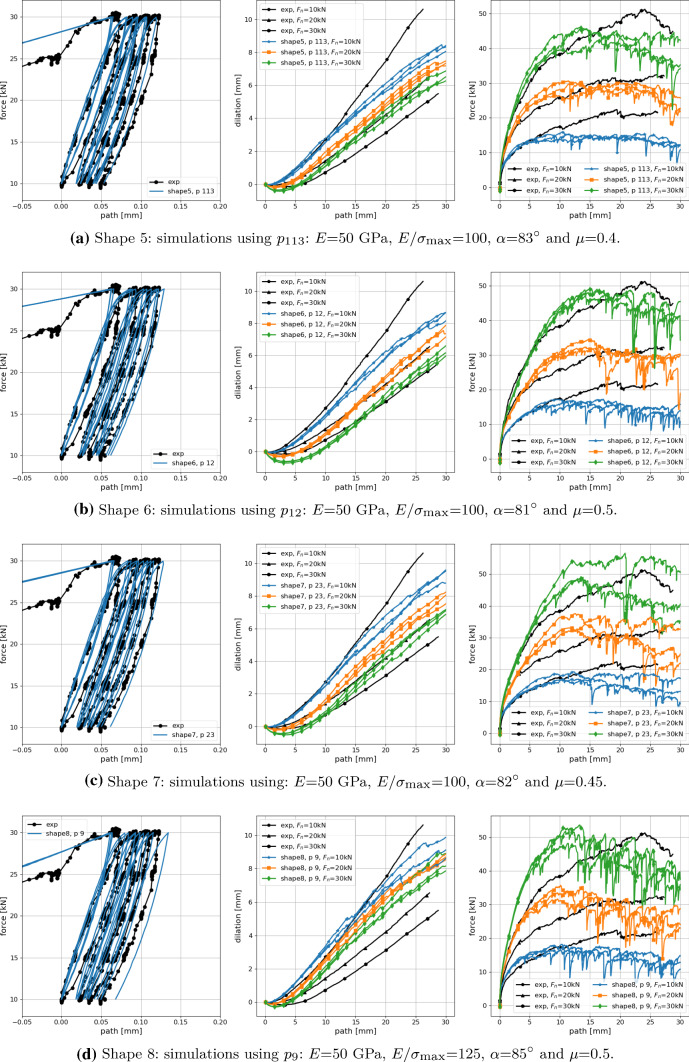
Shapes 5 to 8: results of parametrisation in comparison to Kieselkalk experiments

**Fig. 15:**
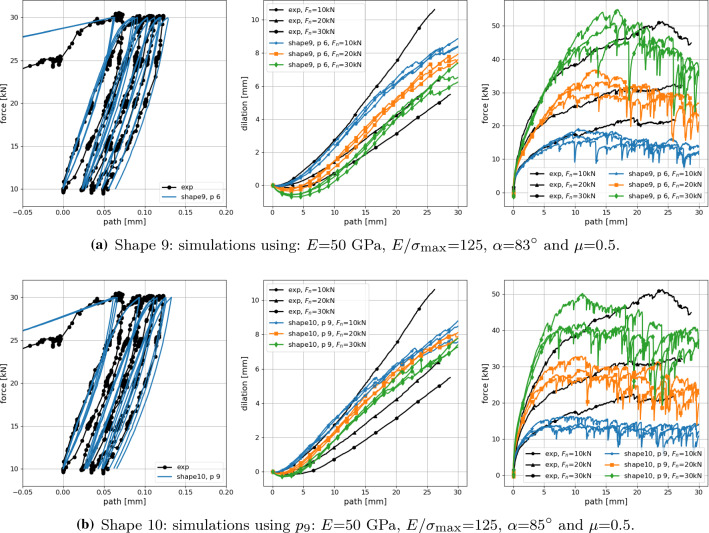
Shapes 9 and 10: results of parametrisation in comparison to Kieselkalk experiments

## Data Availability

The datasets analysed during the current study are openly available in the zenodo.org repository, see [20, 24, 26, 27].

## Appendix: Parametrisation results for shapes 1–10

Below the obtained results of the parametrisation of particle shapes 1–10 will be shown. At first, the scattering of the results will be considered. Figure 12 shows the parameter space used for the parametrisation for Kieselkalk ballast. For each shape, scatter plots for the cost functions $\varepsilon ^{odo}$ and $\varepsilon ^{shear}$ are shown for the three best parameter sets together with the values of the overall cost function $\varepsilon$ for the four best results. Note that the results for shape 7 stem from previous works of the authors and the parameter numbering used differs from the current approach. For all shapes, the obtained values of $\varepsilon ^{odo}$ are mostly lower than those of $\varepsilon ^{shear}$ and show also less scattering. Finally, the results of the simulations using the best parameter set are compared to the experimental results of compression and direct shear tests for Kieselkalk ballast in Figs. 13, 14 and 15.
